# Evaluation of Long-Term Outcomes of Enamel Matrix Derivative in the Treatment of Peri-Implant Disease: A Systematic Review and Meta-Analysis

**DOI:** 10.3390/bioengineering12121296

**Published:** 2025-11-25

**Authors:** Hye-Jung Song, Ki-Jung Jang, Sung-Hoon Han, Na Jin Kim, Won-Jong Park, Jun-Beom Park

**Affiliations:** 1 Graduate School of Clinical Dental Science, The Catholic University of Korea, Seoul 06591, Republic of Korea; hjsong55@catholic.ac.kr; 2 Dental Implantology, Graduate School of Clinical Dental Science, The Catholic University of Korea, Seoul 06591, Republic of Korea; janparq@naver.com; 3 Department of Orthodontics, Seoul St. Mary’s Hospital, College of Medicine, The Catholic University of Korea, Seoul 06591, Republic of Korea; scherazade@hanmail.net; 4 Medical Library, College of Medicine, The Catholic University of Korea, Seoul 06591, Republic of Korea; kimnj@catholic.ac.kr; 5 Department of Oral and Maxillofacial Surgery, Seoul St. Mary’s Hospital, College of Medicine, The Catholic University of Korea, Seoul 06591, Republic of Korea; 6 Department of Periodontics, College of Medicine, The Catholic University of Korea, Seoul 06591, Republic of Korea; 7 Department of Medicine, Graduate School, The Catholic University of Korea, Seoul 06591, Republic of Korea

**Keywords:** dental enamel proteins, peri-implantitis, dental implants

## Abstract

Enamel matrix derivative (EMD) has been proposed as an adjunctive treatment for peri-implantitis, a prevalent complication of dental implants characterized by inflammatory responses and progressive bone loss around the implant site. This study aimed to perform a systematic review and meta-analysis to evaluate the efficacy of EMD compared with control interventions in the treatment of peri-implantitis. A comprehensive literature search was conducted by two independent reviewers using a combination of Medical Subject Headings (MeSHs) and free-text terms. The search included three major electronic databases—Medline via PubMed, the Cochrane Library, and Embase—and studies published up to November 2024. The search aimed to identify relevant clinical trials assessing the long-term outcomes of EMD in peri-implantitis treatment. Out of an initial 54 articles identified, five met the inclusion criteria and were included in the meta-analysis. The pooled mean difference for probing depth reduction with EMD compared to controls was 0.88 (95% confidence of interval (CI): −1.26 to 2.02). The decrease in bleeding on probing yielded a pooled mean difference of 16.70 (95% CI: −4.93 to 38.32). For bone level gain, the pooled mean difference was 0.59 (95% CI: −0.01 to 1.19). This meta-analysis indicated that the most significant improvement with the use of EMD was observed in bone level gain with surgical approach. Moreover, the adjunctive use of EMD in the treatment of peri-implantitis may offer clinical benefits in reducing probing depth and bleeding on probing at the three-month evaluation. These findings support the consideration of EMD as a valuable adjunct in peri-implantitis treatment protocols, warranting further investigation through well-designed, long-term randomized controlled trials.

## 1. Introduction

Enamel matrix proteins, particularly amelogenins, play a crucial role in the formation and regeneration of tooth enamel [1,2]. Building on these biological properties, enamel matrix derivative (EMD) was developed as a therapeutic agent for periodontal regeneration, demonstrating the ability to stimulate the regeneration of key periodontal structures, including cementum, the periodontal ligament, and alveolar bone [3,4]. EMD has been widely used to promote periodontal regeneration and has shown enhanced clinical outcomes, particularly when used in combination with bone grafting materials [5].

Successful dental implant therapy depends not only on osseointegration but also on the maintenance of healthy peri-implant soft and hard tissues [6,7]. EMD has shown promise in promoting bone healing around dental implants due to its regenerative capabilities. Consequently, it has been proposed as a treatment for peri-implantitis—a common complication of dental implants characterized by inflammation and progressive bone loss surrounding the implant fixture [8]. Clinically, peri-implantitis presents similarly to periodontitis, involving destruction of both soft and hard tissue destruction. However, its management often requires distinct strategies, including mechanical debridement, biofilm removal, and adjunctive antimicrobial therapies such as antibiotics, antiseptics, or sustained-release agents [9]. Among these, the adjunctive use of EMD and sustained-release minocycline microspheres has shown promise in improving clinical parameters and reducing Porphyromonas gingivalis levels within three months post-treatment [10].

Although early studies suggest that EMD contribute positively to the surgical management of peri-implantitis [11], the current evidence remains limited. Favorable outcomes have been reported when EMD is used in combination with bone substitutes such as hydroxyapatite, showing improved regenerative potential [12]. Long-term clinical and radiographic follow-ups at three and five years post-surgery have also indicated a positive correlation between EMD application and implant survival rates [13]. Typical surgical protocols include flap elevation, surface decontamination, EMD application, and guided bone regeneration using grafting materials like freeze-dried bone allograft or anorganic bovine bone, often covered with an absorbable membrane or subepithelial connective tissue graft [14].

Despite these encouraging findings, a standardized surgical protocol incorporating EMD remains to be established. To address this gap, the present study aims to systematically review the literature and conduct a meta-analysis to evaluate the clinical outcomes of EMD compared to control interventions in the treatment of peri-implantitis. The null hypothesis states that there is no significant difference in clinical outcomes between treatment with EMD and control approaches.

## 2. Materials and Methods

### 2.1. Protocol

This systematic review was conducted in accordance with the Preferred Reporting Items for Systematic Reviews and Meta-Analyses (PRISMA) guidelines [15]. The review methodology adhered strictly to the PRISMA framework to ensure transparency, reproducibility, and comprehensive reporting. The study protocol was prospectively registered in the PROSPERO database (CRD42024591896). We applied this following structured approach to ensure the selection of high-quality evidence relevant to evaluating the efficacy of EMD in the treatment of peri-implantitis.

### 2.2. Eligibility Criteria

The primary research question guiding this review was: Is there a difference in treatment outcomes associated with the adjunctive use of EMD in patients with peri-implant disease compared to treatments without EMD?

- Participants: Adults diagnosed with peri-implant disease
- Intervention: Adjunctive use of EMD
- Comparison: Control groups receiving treatment without EMD
- Outcomes: Indicators of bone and soft tissue regeneration, including:
  - Change in probing depth
  - Gain in bone level
  - Change in bleeding on probing
  - Change in local plaque index
  - Change in pus discharge
- Study Design: Randomized controlled trials (RCTs) involving adult participants

### 2.3. Inclusion and Exclusion Criteria

- Inclusion Criteria
  - Studies evaluating the surgical treatment of peri-implantitis with adjunctive use of EMD
  - Studies involving mechanical and/or chemical decontamination of implant surfaces
  - RCTs that reported sufficient quantitative data for meta-analysis
- Exclusion Criteria
  - Studies focused exclusively on natural teeth without involvement of dental implants
  - Studies involving patients who had not undergone implant placement or were treated with removable dentures
  - Studies lacking outcome data necessary for meta-analysis

### 2.4. Information Sources and Search Strategy

An experienced information specialist (NJK), affiliated with an academic library, conducted the initial systematic search using a combination of controlled vocabulary (MeSH terms) and free-text keywords to identify relevant studies. The search was performed across three major electronic databases: Medline via PubMed, the Cochrane Database, and Embase, with coverage extending up to 30 November 2024. To ensure the comprehensiveness of the search, two additional reviewers (HJS and KJJ) independently replicated the search using the same methodology, integrating both MeSH terms and free-text terms tailored to each database. In addition to the electronic search, a manual screening of the reference lists of all retrieved full-text articles was performed to identify any potentially eligible studies not captured in the database searches. All references were imported into EndNote reference management software (version 21, Clarivate, Philadelphia, PA, USA), where duplicates were systematically identified and removed. The search strategy was adapted to the syntax and indexing terms specific to each database. Detailed search terms and strategies are provided in Supplementary Material Tables S1 and S2.

### 2.5. Study Selection and Data Extraction

Two reviewers (HJS and KJJ) independently screened the titles and abstracts of all retrieved articles for eligibility, following a blinded assessment process based on the predefined inclusion and exclusion criteria. Any discrepancies between the reviewers were resolved through discussion with a third author (SHH) to reach consensus. Full-text articles of potentially eligible studies were then assessed independently and in duplicate by the same two reviewers to determine their final inclusion. Data extraction was also performed independently using a structured approach based on the PICOS (Population, Intervention, Comparison, Outcomes, and Study design) framework. Extracted data were categorized into the following domains:

- General study characteristics: Author, year of publication, and country of origin
- Participants: Number of patients and treatment sites
- Intervention and comparison: Use of EMD versus control
- Outcomes: Changes in probing depth, gain in bone level, change in bleeding on probing, local plaque index and pus discharge

### 2.6. Risk of Bias Assessment

The methodological quality of the included randomized controlled trials was evaluated using the Cochrane Risk of Bias 2.0 (ROB 2.0) tool. This assessment covered the following domains: the randomization process (selection bias), deviations from intended interventions (performance bias), missing outcome data (attrition bias), measurement of outcomes (detection bias), selection of the reported results (reporting bias), and the overall risk of bias. Each domain, as well as the overall risk of bias for each study, was classified as low risk, some concerns, or high risk. Two reviewers (HJS and KJJ) performed the risk of bias assessments. Any discrepancies were resolved through discussion to reach a consensus.

### 2.7. Data Synthesis and Analysis

The meta-analysis was performed using R software (version 4.3.2; R Project for Statistical Computing). The mean difference (MD) with corresponding 95% confidence intervals (CI) was used as the summary measure for continuous outcomes. A random-effects model was employed to account for potential variability between studies. Statistical significance was set at *p* < 0.05. Heterogeneity among the included studies was evaluated using the *I*^2^ statistic and the tau-square (*τ*^2^) test.

## 3. Results

### 3.1. Study Selection and Data Extraction

**Figure 1:**
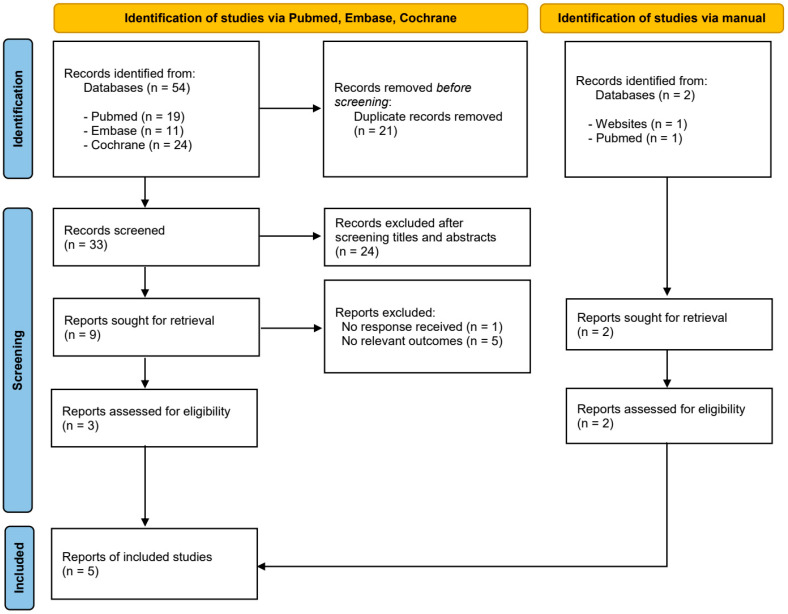
Flowchart illustrating the selection process of studies included in the systematic review.

The initial database search yielded 54 articles. Following the removal of 21 duplicates, the titles and abstracts of the remaining studies were screened, resulting in the exclusion of 23 articles that did not meet the inclusion criteria. A full-text review was conducted for the remaining 33 articles. After applying the eligibility criteria, 24 articles were excluded, and one additional study identified through manual search was included. In total, nine studies were assessed for eligibility. The study selection process is illustrated in the flowchart presented in Figure 1. Details of the excluded full-text articles, along with reasons for exclusion, are provided in Supplementary Material Table S3. Table 1 and Table 2 provide a summary of the main characteristics of the included studies.

### 3.2. Risk of Bias Assessment

**Figure 2:**
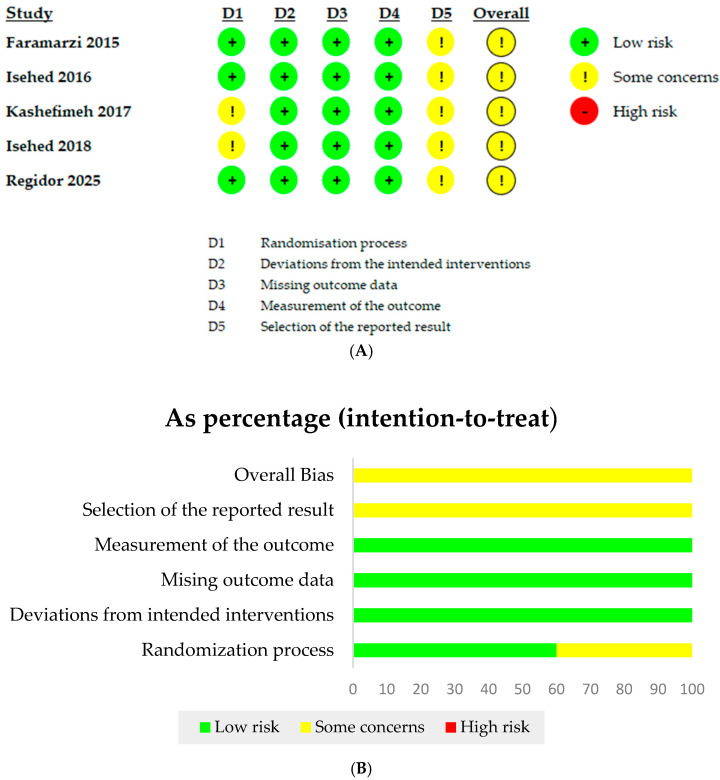
Risk of bias assessment. (**A**) Summary of the risk of bias across individual domains for the included studies. (**B**) Overall risk of bias rating for each study. Faramarzi (2015) [10], Isehed (2016) [16], Kashefimehr (2017) [17], Isehed (2018) [13], and Regidor (2025) [18].

The summary of the risk of bias across individual domains for each included study is presented in Figure 2. This figure visually depicts the risk of bias assessments, with green indicating low risk, yellow indicating some concerns, and red indicating high risk (Figure 2A). A detailed breakdown of the risk of bias evaluations, including domain-specific and overall scores for each study, is provided in Supplementary Material Table S4. All included studies were rated as having some concerns regarding the overall risk of bias. Notably, areas such as the randomization process and the selection of reported outcomes were common sources of potential bias, warranting cautious interpretation of the findings (Figure 2B).

### 3.3. Meta-Analysis

Five studies—Faramarzi (2015) [10], Isehed (2016) [16], Kashefimehr (2017) [17], Isehed (2018) [13], and Regidor (2025) [18]—were included in the meta-analysis to evaluate the efficacy of adjunctive use of EMD in the treatment of peri-implantitis.

#### 3.3.1. Change in Probing Depth

**Figure 3:**
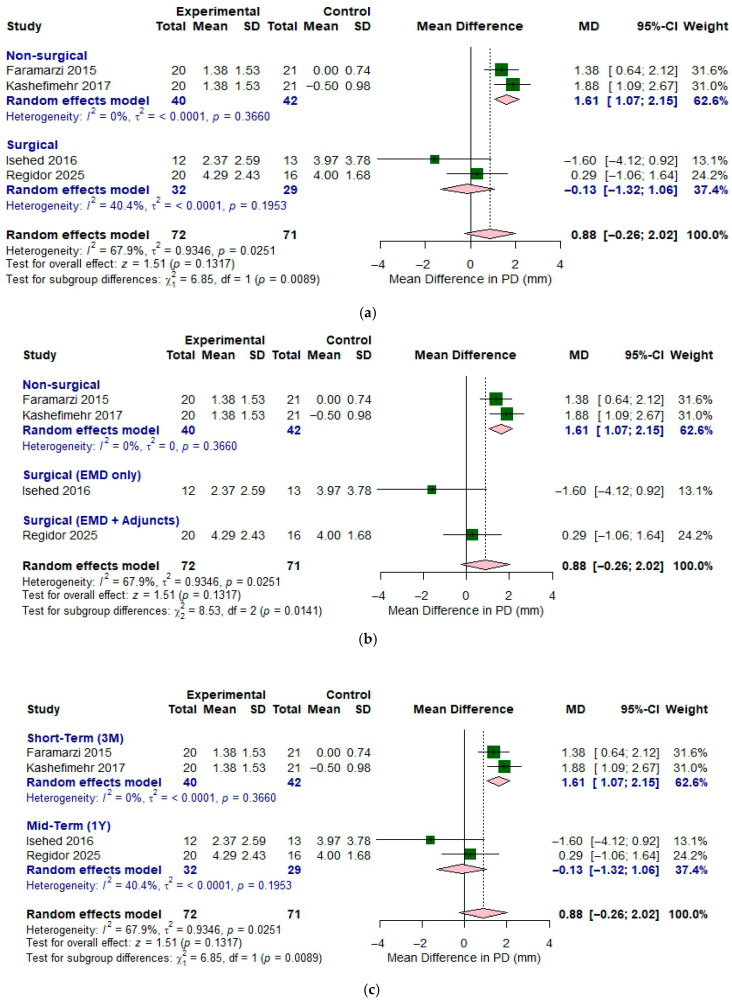
Forest plot comparing the effect of enamel matrix derivative versus control on probing depth change. (**a**) Comparing the effect of non-surgical and surgical approaches. (**b**) Different types of surgical approaches. (**c**) Different follow-up periods. Included studies: Faramarzi 2015 [10], Isehed 2016 [16], Kashefimehr 2017 [17], and Regidor 2025 [18].

Figure 3a shows forest plot illustrating the effect of EMD with different treatment protocol on change in probing depth. The analysis was conducted in two subgroups: non-surgical and surgical approaches. For the non-surgical approach, two studies (Faramarzi (2015) [10] and Kashefimehr (2017) [17]) were used. This subgroup demonstrated substantial heterogeneity (*I*^2^ = 0%), indicating low heterogeneity between the studies. The pooled MD was 1.61 (95% CI of 1.07 to 2.15) and the forest plot indicated a trend favoring adjunctive use of EMD for greater probing depth reduction. In the surgical subgroup, moderate heterogeneity was observed (*I*^2^ = 40.4%, *p* > 0.05), indicating some inconsistency among study results. For surgical approach, two studies (Isehed (2016) [16] and Regidor (2025) [18]) were analyzed. The use of EMD demonstrated a pooled MD of 0.88 (95% CI of −0.26 to 2.02), implying no apparent differences between two groups. The application of EMD showed a pooled MD of −1.60 (95% CI of −4.12 to 0.92) and the combination of EMD and adjuncts led to a pooled MD of 0.29 (95% CI of −1.06 to 1.64). When considering all four included studies together (total sample size: experimental *n* = 72, control *n* = 71), substantial heterogeneity was observed (*I*^2^ = 67.9%, *p* < 0.05), indicating considerable variability between studies. The overall effect demonstrated a pooled MD of 0.88 (95% CI of −0.26 to 2.02), with a test for overall effect yielding z = 1.51 (*p* = 0.1317). This suggests a positive trend toward improved outcomes with EMD, though the effect did not reach statistical significance at the pooled level.

The results of different follow-up periods are shown in Figure 3c. A random effects model was applied for the short-term of three- months follow-up evaluation, and the *I*^2^ value (67.9%) indicated considerable variability between studies. The subgroup analysis on 3 months showed that the pooled MD was of 1.61 (95% CI of 1.07 to 2.15) and the forest plot indicated a trend favoring the adjunctive use of EMD for greater probing depth reduction. Subgroup on analysis at one year demonstrated an *I*^2^ value of 40.4% and *p*-value of 0.1953. The application of EMD showed a pooled MD of –0.13 (95% CI of −1.32 to 1.06) on one-year follow up evaluation.

#### 3.3.2. Decrease in Bleeding on Probing

**Figure 4:**
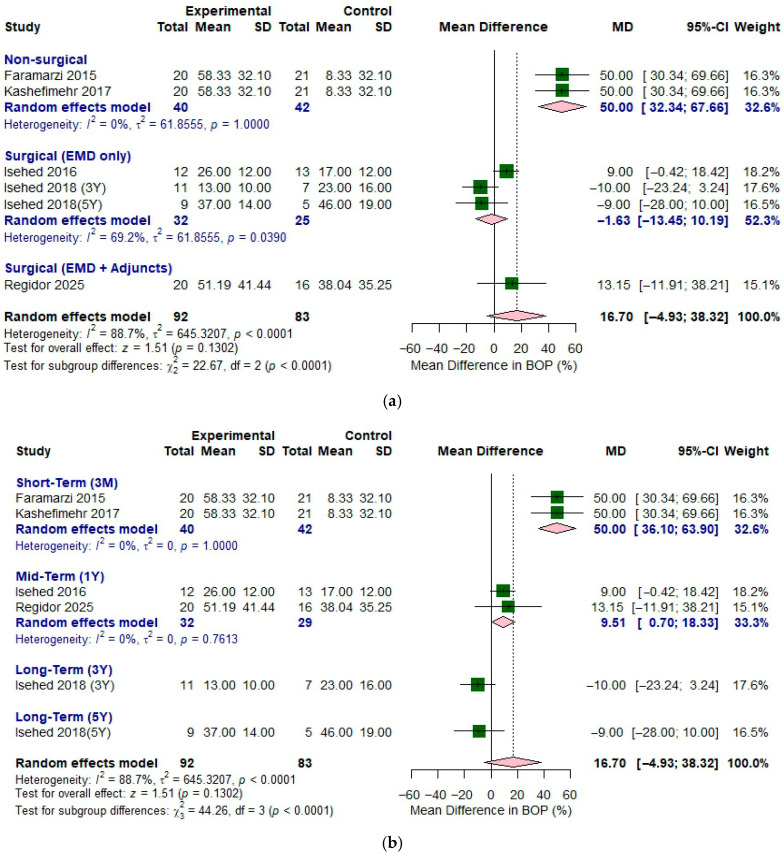
Forest plot comparing enamel matrix derivative versus control for the reduction in bleeding on probing in the treatment of peri-implantitis. (**a**) Different types of non-surgical and surgical approaches. (**b**) Different follow-up periods. Included studies: Faramarzi 2015 [10], Isehed 2018 [13], Isehed 2016 [16], Kashefimehr 2017 [17], and Regidor 2025 [18].

Figure 4a shows a forest plot illustrating the effect of EMD with different treatment protocols on decrease in bleeding on probing. The analysis was conducted in two subgroups: non-surgical and surgical approaches. For non-surgical approaches, two studies (Faramarzi (2015) [10] and Kashefimehr (2017) [17]) were used. This subgroup demonstrated low heterogeneity (*I*^2^ = 0%, *p* = 1.0). The pooled MD was 50.0 (95% CI of 32.34 to 67.66) and the forest plot indicated a trend favoring the adjunctive use of EMD for greater decrease in bleeding on probing.

For surgical approach, three studies—Isehed (2016) [16], Isehed (2018) [13], and Regidor (2025) [18]) were used for the analysis. In the surgical subgroup of EMD only group, substantial heterogeneity was observed (*I*^2^ = 69.2%, *p* < 0.05), indicating considerable variability exists between studies. The pooled MD was −1.63 (95% CI of −13.45 to 10.19) and the forest plot indicated no significant difference between the groups regarding the decrease in bleeding on probing.

When considering all five included studies together (total sample size: experimental *n* = 92, control *n* = 83), substantial heterogeneity was observed (*I*^2^ = 88.7%, *p* < 0.01), indicating marked variability between the studies. The overall effect demonstrated a pooled MD of 16.70 (95% CI of −4.93 to 38.32), with a test for overall effect yielding z = 1.51 (*p* = 0.1302). This suggests a positive trend toward improved outcomes with EMD, though the effect did not reach statistical significance at the pooled level.

The results of different follow-up periods are shown in Figure 4b. A random effects model was applied for short-term of three months follow-up evaluation, and *I*^2^ value (0.0%, *p* = 1.0) indicated that low heterogeneity between the studies. Subgroup on 3 months showed that the pooled MD was 50.0 (95% CI of 36.1 to 63.9) and the forest plot indicated a trend favoring the adjunctive use of EMD for greater reduction in bleeding on probing. Subgroup on one year demonstrated *I*^2^ value of 0.0% and the *p*-value of 0.7613. The application of EMD showed a pooled MD of 9.51 (95% CI of 0.70 to 18.33) on one-year follow up evaluation, showing greater reduction in bleeding on probing with the application of EMD.

#### 3.3.3. Increase in the Bone Level

**Figure 5:**
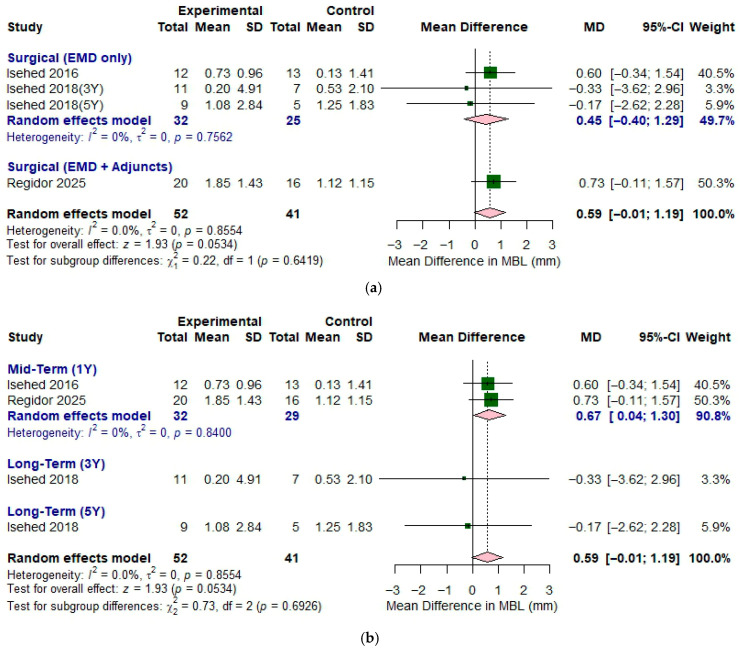
Forest plot illustrating the comparison between enamel matrix derivative and control groups for bone level gain in the treatment of peri-implantitis across various follow-up periods. (**a**) Different types of non-surgical and surgical approaches. (**b**) Different follow-up periods. Included studies: Isehed 2018 [13], Isehed 2016 [16], and Regidor 2025 [18].

Figure 5a shows forest plot illustrating the effect of EMD with different treatment protocol on bone level gain. For the surgical subgroup of EMD only group, two studies—Isehed (2016) [16] and Isehed (2018) [13] were used for the analysis. *I*^2^ value (0.0%, *p* = 0.7562) indicated that low heterogeneity between the studies. The pooled MD was 0.45 (95% CI of −0.40 to 1.29) and the forest plot indicated that there was no significant difference between the groups regarding the bone level gain. When considering all three included studies together (total sample size: experimental *n* = 52, control *n* = 41), low heterogeneity was observed (*I*^2^ = 0.0%, *p* = 0.8554). The overall effect demonstrated a pooled MD of 0.59 (95% CI of −0.01 to 1.19), with a test for overall effect yielding z = 1.93 (*p* = 0.05). This suggests a positive trend toward improved outcomes with EMD, indicating greater bone level gain with the application of EMD.

The results of different follow-up periods are shown in Figure 5b. For mid-term of one year follow-up evaluation, *I*^2^ value (0.0%, *p* = 0.84) indicated that low heterogeneity between the studies. Subgroup on one year showed that the pooled MD was 0.67 (95% CI of 0.04 to 1.30) and the forest plot indicated a trend favoring the adjunctive use of EMD for greater bone level gain.

### 3.4. Publication Bias

**Figure 6:**
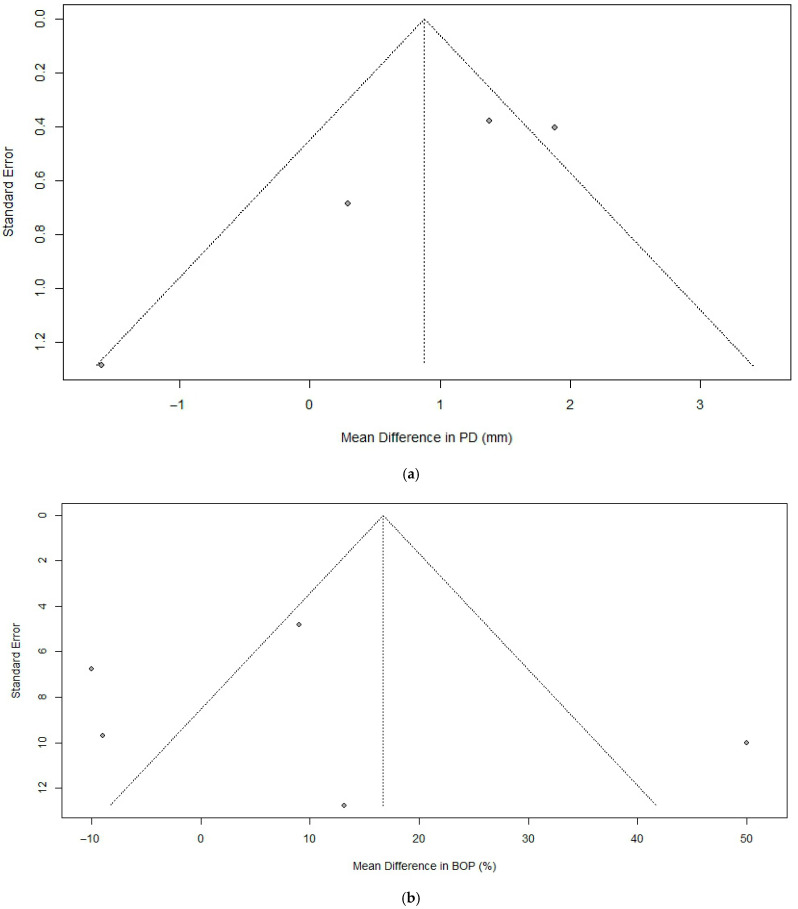

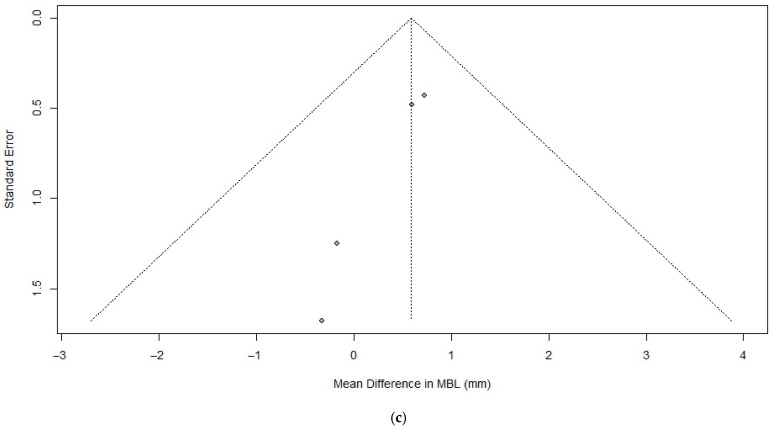
Funnel plots illustrating the assessment of publication bias for each outcome measure: (**a**) Change in probing depth. (**b**) Reduction in bleeding on probing. (**c**) Bone level gain.

The results of the analyses for publication bias are shown in Figure 6 and Table 3. The analysis for publication bias, assessed through original and trim-and-fill methods along with Egger’s regression test, revealed varying degrees of potential bias across the evaluated variables. For probing depth, two additional studies were added in the trim-and-fill analysis, and Egger’s test indicated no significant publication bias (*p* = 0.07). Similarly, for bleeding on probing, one additional study was added in the trim-and-fill analysis, and Egger’s test indicated no significant publication bias (*p* = 0.44). In contrast, for marginal bone level, two additional studies were added in the trim-and-fill analysis and Egger’s regression showed a statistically significant result (*p* = 0.02). Overall, while most variables did not show evidence of publication bias, the findings for marginal bone level suggest the need for cautious interpretation.

## 4. Discussion

This systematic review and meta-analysis evaluated the long-term efficacy of EMD in the management of peri-implant disease. Although EMD consistently showed a trend toward improved clinical and radiographic outcomes, most differences did not reach statistical significance. Notably, EMD demonstrated a significant benefit in bone level gain, suggesting its potential role for enhancing regeneration around the implant.

The long-term outcomes presented in this study indicate that EMD contribute positively to both clinical and radiographic parameters in the treatment of peri-implant disease. While non-surgical interventions alone have shown limited effects on clinical parameters and biomarker levels, adjunctive treatments including EMD have demonstrated more favorable short-term outcomes [19]. In this study, the significant clinical benefits of EMD in reducing probing depth and bleeding on probing were observed at three months. Moreover, surgical therapies incorporating EMD appeared to yield greater improvements in inflammation control and clinical indicators over both the short and long term when compared with non-surgical approaches [11]. This study clearly demonstrated the benefits of EMD in bone level gain when using a surgical approach. Combination therapies involving EMD have also shown promising success rates. One study reported a 56.6% success rate after 36 months of treatment using EMD as part of a combination therapy, supporting its effectiveness in managing peri-implantitis [20]. Furthermore, a randomized controlled trial reported a 100% implant survival rate in the EMD group at three years and 85% at five years, compared to 83% and 75%, respectively, in the non-EMD group [13].

The therapeutic effects of EMD in the treatment of peri-implant disease can be attributed to its multifaceted influence on both inflammatory modulation and tissue regeneration. Adjunctive use of EMD has been associated with a reduction in key inflammatory biomarkers in peri-implant crevicular fluid, indicating a more favorable local immune environment following treatment [19]. Specifically, EMD has been shown to downregulate pro-inflammatory cytokines, including interleukin (IL)-1β, IL-6, IL-17, and receptor activator of nuclear factor-kappa B ligand, which are critical mediators of peri-implant inflammation and bone resorption [17,21]. In addition, EMD promotes the expression of leukocyte chemotactic factors and adhesion molecules in vascular endothelial cells, enhancing immune cell recruitment and function at the site of inflammation [22]. Furthermore, EMD facilitates immune modulation by enhancing the proliferation and migration of T lymphocytes, inducing monocyte differentiation, and promoting the clearance of bacteria and tissue debris [21]. A notable shift toward a Gram-positive and aerobic bacterial profile has also been observed following EMD application, potentially contributing to a more stable and healing-conducive microbiome in peri-implant tissues [16].

At the cellular level, EMD exerts a stimulatory effect on various cell types involved in tissue regeneration. It has been reported to enhance the attachment, proliferation, and migration of fibroblasts, osteoblasts, and other regenerative cells, processes that are critical for effective wound healing [23]. EMD also supports osteoblast differentiation and upregulates the expression of bone-related proteins such as osteocalcin and transforming growth factor-beta 1, both of which play essential roles in bone remodeling and regeneration [24]. Moreover, the regenerative effects of EMD appear to be mediated by several intracellular signaling pathways, including the extracellular signal-regulated kinase 1/2, c-Jun N-terminal kinase, and p38 mitogen-activated protein kinase pathways [25,26]. These pathways are known to regulate cell proliferation, migration, and differentiation, thereby contributing to the observed clinical improvements associated with EMD treatment. Collectively, these mechanisms highlight the biological potential of EMD as a therapeutic agent that addresses both inflammation and tissue regeneration in the management of peri-implant disease.

Variations in treatment protocols across clinical settings may influence the application of EMD. The recommended amount of EMD typically depends on the size and configuration of the defect, with clinicians generally applying it as a thin, uniform layer to ensure adequate coverage of the affected area. The exact volume used is determined by the clinical context and the discretion of the treating dentist or periodontist. Additionally, the carrier system for EMD can vary depending on the manufacturer and product formulation [27,28]. Common carriers include hyaluronic acid, propylene glycol, glycerin, and water, which aid in the delivery of EMD by providing suitable consistency and handling properties for clinical use [29]. Notably, the adsorption of EMD onto grafting materials can vary significantly based on the carrier employed [27]. In regenerative procedures, EMD is often combined with scaffold materials to support and enhance periodontal tissue regeneration.

Despite the rigorous methodology employed in this meta-analysis—including a comprehensive literature search and strict adherence to PRISMA guidelines—several limitations should be acknowledged. A major source of concern lies in the methodological quality of the studies included in both the qualitative and quantitative syntheses [30]. The nature of the intervention, which involved the adjunctive application of a gel, likely rendered participant blinding unfeasible [31,32], potentially introducing performance bias. Furthermore, selective outcome reporting in some studies might have affected the reliability and generalizability of the results. The statistical analysis also revealed substantial heterogeneity, indicating possible underlying differences in study design, patient populations, and treatment protocols. Such heterogeneity limits the external validity of the pooled estimates and warrants cautious interpretation. Given the limited number of included studies, the statistical power of Egger’s regression test to detect publication bias might have been insufficient, and the observed funnel plot asymmetry could have been influenced by factors other than publication bias. These findings highlight the need for well-designed randomized controlled trials with clearly defined inclusion criteria to confirm the efficacy of the intervention [33]. Future studies should also consider standardizing follow-up durations and accounting for variability in patient characteristics to enhance clinical applicability.

Collectively, the results of this meta-analysis suggest that EMD is associated with more favorable outcomes in terms of probing depth reduction, improvements in bleeding on probing and bone level gain. Overall, these findings support the potential clinical advantage of EMD in the management of peri-implantitis and suggest that it may merit prioritized consideration in appropriate treatment protocols.

## 5. Conclusions

This meta-analysis indicates that the adjunctive use of EMD in the treatment of peri-implantitis may offer clinical benefits. The most significant improvement with the use of EMD was observed in bone level gain with surgical approach. Moreover, the adjunctive use of EMD in the treatment of peri-implantitis can offer clinical benefits in reducing probing depth and bleeding on probing at the three-month evaluation. These findings support the consideration of EMD as a valuable adjunct in peri-implantitis treatment protocols, warranting further investigation through well-designed, long-term randomized controlled trials.

## Figures and Tables

**Table 1:** Design of the included studies.

Study (Author, Year)	Country	Study Design	Treatment Modality	Inclusion Criteria	Sample Size (Test: Control)
Faramarzi et al., (2015) [10]	Iran	RCT (double-blind, three-arm parallel group)	Non-surgical	Adults (≥18) with at least one implant functional ≥ 1 year Peri-implant mucositis/mild peri-implantitis BOP, PD ≥ 4 mm No soft tissue recession Radiographic bone loss ≤ 2 mm Classified as Implant Success Index Grade III/IV	20:21
Isehed et al., (2016) [16]	Sweden	RCT (double-blind, parallel group)	Surgical	Patients with peri-implantitis Pocket depth ≥ 5 mm BOP and/or suppuration (pus) Angular bone loss ≥ 3 mm at ≥1 implant (on radiograph)	12:13
Kashefimehr et al., (2017) [17]	Iran	RCT (double-blind)	Non-surgical	Adults (≥18) with implant functional ≥ 1 year Severe peri-implant mucositis or mild peri-implantitis Excluded if PD ≥ 6 mm at baseline	20:21
Isehed et al., (2018) [13]	Sweden	RCT (double-blind, parallel groups)	Surgical	Same as 2016 (extended follow-up of 2016)	3 years: 11:7 5 years: 9:5
Regidor et al., (2025) [18]	Spain	RCT (two parallel groups)	Surgical	Adults (≥18) with implant functional ≥ 1 year Severe peri-implantitis defined as: PPD ≥ 5 mm Persistent BOP and/or SOP despite prior non-surgical therapy Intraosseous defect ≥ 3 mm depth and ≤4 mm width	20:16 (ESS) *

ESS, effective sample size; BOP, bleeding on probing; PD, probing depth; SOP, suppuration on probing. * Patient ESS = Number of patients ÷ [1 + (average implant number − 1) × intracluster correlation coefficient].

**Table 2:** Main characteristics of the included studies.

Study (Author, Year)	Test Procedure	Control Procedure	Follow-Up	Outcomes
Faramarzi et al., (2015) [10]	Mechanical debridement (MD) followed by application of EMD	Mechanical subgingival debridement only	2 weeks, 3 months	PD, BOP
Isehed et al., (2016) [16]	Flap surgery with debridement and saline cleaning, followed by application of EMD.	Same surgical procedure without EMD.	12 months	MBL change, PD, BOP, plaque index, suppuration (pus), microbiological composition, implant survival/loss
Kashefimehr et al., (2017) [17]	Subgingival debridement with ultrasonic device and air polishing, followed by application of EMD	Subgingival debridement with ultrasonic device and glycine-based air polishing only	3 months	BOP, PD change, pain on probing, plaque index
Isehed et al., (2018) [13]	Flap surgery with debridement and saline cleaning, followed by application of EMD.	Same surgical procedure without EMD	1, 3, and 5 years	Implant survival, MBL change BOP, plaque index, suppuration (pus)
Regidor et al., (2025) [18]	Flap surgery, implant surface decontamination, bone graft + resorbable membrane + EMD	Flap surgery, implant surface decontamination, bone graft + resorbable membrane (without EMD)	6 months	PD change, BOP, suppuration (SOP), recession, keratinized mucosa, MBL change, early healing index
			12 months	

PD, probing depth; BOP, bleeding on probing; MBL, marginal bone level; SOP, suppuration on probing.

**Table 3:** Analyses for publication bias.

Variables	Original Analysis		Trim-and-Fill Analysis		Egger’s Regression Test			
	MD (95% CI)	*p*-Value	MD (95% CI)	Added Studies/Total Studies	t-Value	df	*p*-Value	Bias Estimate
PD	0.88	0.13	1.62	2/6	−3.59	2	0.07	−3.71
	(−0.26 to 2.02)		(0.18 to 3.06)					(SE = 1.03)
BOP	16.70	0.13	9.41	1/7	0.86	4	0.44	3.21
	(−4.93 to 38.32)		(−13.95 to 36.77)					(SE = 3.74)
MBL	0.59	0.05	0.67	2/6	−7.03	2	0.02	−0.93
	(−0.01 to 1.19)		(0.10 to 1.24)					(SE = 0.13)

PD, probing depth; BOP, bleeding on probing; MBL, marginal bone level.

## Data Availability

The original contributions presented in the study are included in the article/Supplementary Materials, further inquiries can be directed to the corresponding authors.

## Supplementary Materials

The following supporting information can be downloaded at: https://www.mdpi.com/article/10.3390/bioengineering12121296/s1, Table S1. Search strategy of the online databases; Table S2. Manual searches; Table S3. Excluded studies from full-text reading; Table S4. Risk of bias. References [10,13,16,17,18] are cited in the Supplementary Materials.

## References

[1] Ozbay D., Tunc S., Uraz Çörekci A., Guler Ayyildiz B., Cula S. (2024). The Evaluation of Enamel Matrix Derivative on the Bone Regenerative Potential of the Dental Implant with the Transcrestal Sinus Floor Elevation Approach: A Randomized, Parallel CBCT Study. Int. J. Oral Maxillofac. Implant..

[2] Nakayama Y., Ogihara-Takeda M., Saito Y., Yamaguchi A., Ogata Y. (2024). Early wound healing at 1 week postoperatively in periodontal tissue regeneration therapy: Enamel matrix derivative versus recombinant human fibroblast growth factor. J. Periodontal Implant Sci..

[3] Na K.H., Lee H.J., Lee J.E., Park J.B. (2024). Regeneration of rabbit calvarial defects with combination of stem cells and enamel matrix derivative: A microcomputed tomography and histological evaluation comparing two- and three-dimensional cell constructs. Medicina.

[4] Nakayama Y., Tabe S., Igarashi K., Moriya S., Katsumata T., Kobayashi R., Nakagawa S., Nishino T., Fukuoka N., Hosono K. (2024). Comparison of early wound healing using modified papilla preservation technique between enamel matrix derivative and recombinant human fibroblast growth factor. J. Periodontal Implant Sci..

[5] Li W., Xiao L., Hu J. (2012). The use of enamel matrix derivative alone versus in combination with bone grafts to treat patients with periodontal intrabony defects: A meta-analysis. J. Am. Dent. Assoc..

[6] Kim Y.J., Song Y.W., Park S.Y., Cha J.K., Lee H.J., Yang S.M., Park J.B., Koo K.T. (2024). Current understanding of the etiology, diagnosis, treatment, and management of peri-implant diseases: A narrative review for the consensus report of the Korean Academy of Periodontology. J. Periodontal Implant Sci..

[7] Lee H.K., Hong J.Y., Shin S.I., Herr Y., Lim H.C., Chung J.H. (2024). Soft-tissue volume augmentation using a connective tissue graft and a volume-stable collagen matrix with polydeoxyribonucleotide for immediate implant placement: A pilot study in a dog model. J. Periodontal Implant Sci..

[8] Roccuzzo A., Imber J.C., Salvi G.E., Roccuzzo M. (2023). Peri-implantitis as the consequence of errors in implant therapy. Periodontol 2000.

[9] Lasserre J.F., Brecx M.C., Toma S. (2018). Oral Microbes, Biofilms and Their Role in Periodontal and Peri-Implant Diseases. Materials.

[10] Faramarzi M., Goharfar Z., Pourabbas R., Kashefimehr A., Shirmohmmadi A. (2015). Microbiological and clinical effects of enamel matrix derivative and sustained-release micro-spherical minocycline application as an adjunct to non-surgical therapy in peri-implant mucosal inflammation. J. Korean Assoc. Oral Maxillofac. Surg..

[11] Moldovan R., Mester A., Piciu A., Bran S., Onisor F. (2022). Clinical Outcomes of Enamel Matrix Derivate Used in Surgical and Non-Surgical Treatment of Peri-Implantitis: A Systematic Review of Clinical Studies. Medicina.

[12] Pilenza D., Filippi A., Walter C., Zitzmann N.U., Bornstein M.M., Kühl S. (2022). Surgical therapy of peri-implantitis with adjunctive hydroxyapatite and enamel matrix derivative: A 1-year retrospective case series. Swiss Dent. J..

[13] Isehed C., Svenson B., Lundberg P., Holmlund A. (2018). Surgical treatment of peri-implantitis using enamel matrix derivative, an RCT: 3- and 5-year follow-up. J. Clin. Periodontol..

[14] Froum S.J., Froum S.H., Rosen P.S. (2015). A Regenerative Approach to the Successful Treatment of Peri-implantitis: A Consecutive Series of 170 Implants in 100 Patients with 2- to 10-Year Follow-up. Int. J. Periodontics Restor. Dent..

[15] Page M.J., McKenzie J.E., Bossuyt P.M., Boutron I., Hoffmann T.C., Mulrow C.D., Shamseer L., Tetzlaff J.M., Akl E.A., Brennan S.E. (2021). The PRISMA 2020 statement: An updated guideline for reporting systematic reviews. Syst. Rev..

[16] Isehed C., Holmlund A., Renvert S., Svenson B., Johansson I., Lundberg P. (2016). Effectiveness of enamel matrix derivative on the clinical and microbiological outcomes following surgical regenerative treatment of peri-implantitis. A randomized controlled trial. J. Clin. Periodontol..

[17] Kashefimehr A., Pourabbas R., Faramarzi M., Zarandi A., Moradi A., Tenenbaum H.C., Azarpazhooh A. (2017). Effects of enamel matrix derivative on non-surgical management of peri-implant mucositis: A double-blind randomized clinical trial. Clin. Oral Investig..

[18] Regidor E., Dionigi C., Ghoraishi M., Salazar J., Trullenque-Eriksson A., Derks J., Ortiz-Vigón A. (2025). Enamel Matrix Derivative in the Reconstructive Surgical Therapy of Peri-Implantitis: A Randomized Clinical Trial. J. Periodontal Res..

[19] Moaven H., Giacaman A., Beltrán V., Sam Y.H., Betancur D., Mainas G., Tarjomani S.A., Donos N., Sousa V. (2022). Biomarker Expression of Peri-Implantitis Lesions before and after Treatment: A Systematic Review. Int. J. Environ. Res. Public Health.

[20] Mercado F., Hamlet S., Ivanovski S. (2018). Regenerative surgical therapy for peri-implantitis using deproteinized bovine bone mineral with 10% collagen, enamel matrix derivative and Doxycycline-A prospective 3-year cohort study. Clin. Oral Implant. Res..

[21] Miron R.J., Dard M., Weinreb M. (2015). Enamel matrix derivative, inflammation and soft tissue wound healing. J. Periodontal Res..

[22] Yaita N., Maruyama K., Hiroyasu K., Sato S. (2025). Immunogenic effects of enamel matrix derivative on human alveolar ridge mucosa-derived vascular endothelial cells under lipopolysaccharide stimulation. Odontology.

[23] Bosshardt D.D. (2008). Biological mediators and periodontal regeneration: A review of enamel matrix proteins at the cellular and molecular levels. J. Clin. Periodontol..

[24] Lyngstadaas S.P., Wohlfahrt J.C., Brookes S.J., Paine M.L., Snead M.L., Reseland J.E. (2009). Enamel matrix proteins; old molecules for new applications. Orthod. Craniofac. Res..

[25] Ge C., Xiao G., Jiang D., Franceschi R.T. (2007). Critical role of the extracellular signal-regulated kinase-MAPK pathway in osteoblast differentiation and skeletal development. J. Cell Biol..

[26] Greenblatt M.B., Shim J.H., Bok S., Kim J.M. (2022). The Extracellular Signal-Regulated Kinase Mitogen-Activated Protein Kinase Pathway in Osteoblasts. J. Bone Metab..

[27] Miron R.J., Bosshardt D.D., Buser D., Zhang Y., Tugulu S., Gemperli A., Dard M., Caluseru O.M., Chandad F., Sculean A. (2015). Comparison of the capacity of enamel matrix derivative gel and enamel matrix derivative in liquid formulation to adsorb to bone grafting materials. J. Periodontol..

[28] Apicella A., Heunemann P., Bolisetty S., Marascio M., Gemperli Graf A., Garamszegi L., Mezzenga R., Fischer P., Plummer C.J., Månson J.A. (2015). The Influence of Arginine on the Response of Enamel Matrix Derivative (EMD) Proteins to Thermal Stress: Towards Improving the Stability of EMD-Based Products. PLoS ONE.

[29] Gestrelius S., Andersson C., Johansson A.C., Persson E., Brodin A., Rydhag L., Hammarström L. (1997). Formulation of enamel matrix derivative for surface coating: Kinetics and cell colonization. J. Clin. Periodontol..

[30] Suhandi C., Mohammed A.F.A., Wilar G., El-Rayyes A., Wathoni N. (2023). Effectiveness of Mesenchymal Stem Cell Secretome on Wound Healing: A Systematic Review and Meta-analysis. Tissue Eng. Regen. Med..

[31] Karanicolas P.J., Farrokhyar F., Bhandari M. (2010). Practical tips for surgical research: Blinding: Who, what, when, why, how?. Can. J. Surg..

[32] Wartolowska K., Beard D., Carr A. (2017). Blinding in trials of interventional procedures is possible and worthwhile. F1000Res.

[33] Zabor E.C., Kaizer A.M., Hobbs B.P. (2020). Randomized Controlled Trials. Chest.

