# Supplementary material for: Evaluation of Long-Term Outcomes of Enamel Matrix Derivative in the Treatment of Peri-Implant Disease: A Systematic Review and Meta-Analysis
Source: Bioengineering (Basel). 2025 Nov 25;12(12):1296. doi: 10.3390/bioengineering12121296 (PMC12729878; doi:10.3390/bioengineering12121296)
Supplement: Supplementary file 1 [file bioengineering-12-01296-s001.zip › bioengineering-3963175-supplementary.pdf]

Supplementary Table S1. Search strategy of the online databases.

Databases	Search	Search strategy
PubMed	#1	"Peri-Implantitis"[Mesh]
	#2	"Peri-Implantitis"[TW] OR "Peri-Implantitides"[TW] OR "Peri Implantitis"[TW] OR "Periimplantitis"[TW] OR "Periimplantitides"[TW] OR "peri-implant disease"[TW] OR "peri-implant diseases"[TW] OR "Peri-implant"[TW] OR "peri-implant mucositis"[TW]
	#3	"Dental Implants"[Mesh]
	#4	"Dental Implants"[TW] OR "Dental Implant"[TW] OR "Implant, Dental"[TW] OR "Implants, Dental"[TW] OR "Dental Prostheses, Surgical"[TW] OR "Prostheses, Surgical Dental"[TW] OR "Prosthesis, Surgical Dental"[TW] OR "Surgical Dental Prostheses"[TW] OR "Surgical Dental Prosthesis"[TW] OR "Dental Prosthesis, Surgical"[TW] OR "Dental Implants, Mini"[TW] OR "Dental Implant, Mini"[TW] OR "Mini Dental Implant"[TW] OR "Mini Dental Implants"[TW]
	#5	"Dental Implantation"[Mesh]
	#6	"Dental Implantation"[TW] OR "Implantation, Dental"[TW] OR "Implantation, Dental Prosthesis"[TW] OR "Dental Prosthesis Implantations"[TW] OR "Prosthesis Implantation, Dental"[TW] OR "Dental Prosthesis Implantation"[TW] OR "Dental Implant Therapy"[TW] OR "Dental Implant Therapies"[TW] OR "Implant Therapy, Dental"[TW] OR "Therapy, Dental Implant"[TW]
	#7 Combine	#1 OR #2 OR #3 OR #4 OR #5 OR #6
	#8	"enamel matrix proteins" [Supplementary Concept]
	#9	"enamel matrix proteins"[TW] OR "enamel matrix derivatives"[TW] OR "EMDOGAIN"[TW] OR "enamel matrix derivative"[TW]
	#10	"Dental Enamel Proteins"[Mesh]
	#11	"Dental Enamel Proteins"[TW] OR "Enamel Proteins, Dental"[TW] OR "Proteins, Dental Enamel"[TW]
	#12 Combine	#8 OR #9 OR #10 OR #11
	#13 Combine	#7 AND #12
	#14 Limit	#13 AND ((randomizedcontrolledtrial[Filter]) OR ("Randomized Controlled Trial" [Publication Type] OR "Controlled Clinical Trial" [Publication Type] OR "Randomized Controlled Trials as Topic"[Mesh] OR "Random Allocation"[Mesh] OR "Double-Blind Method"[Mesh] OR "Single-Blind Method"[Mesh] OR "Clinical Trial" [Publication Type] OR "Clinical Trials as Topic"[Mesh] OR "Clinical Trial"[TW] OR ((singl*[TW] OR doubl*[TW] OR trebl*[TW] OR tripl*[TW]) AND (mask*[TW] OR blind*[TW])) OR "Placebos"[Mesh] OR placebo*[TW] OR random*[TW] OR "Research Design"[Mesh:NoExp]) NOT ("Animals"[Mesh] NOT "Humans"[Mesh]))
EMBASE	#1	"periimplantitis"/exp
	#2	"Peri-Implantitis":ti,ab,kw,de OR "Peri-Implantitides":ti,ab,kw,de OR "Peri Implantitis":ti,ab,kw,de OR "Periimplantitis":ti,ab,kw,de OR "Periimplantitides":ti,ab,kw,de OR "peri-implant disease":ti,ab,kw,de OR "peri-implant diseases":ti,ab,kw,de OR "Peri-implant":ti,ab,kw,de OR "peri-implant mucositis":ti,ab,kw,de
	#3	"tooth implant"/exp

	#4	"Dental Implants":ti,ab,kw,de OR "Dental Implant":ti,ab,kw,de OR "Implant, Dental":ti,ab,kw,de OR "Implants, Dental":ti,ab,kw,de OR "Dental Prostheses, Surgical":ti,ab,kw,de OR "Prostheses, Surgical Dental":ti,ab,kw,de OR "Prosthesis, Surgical Dental":ti,ab,kw,de OR "Surgical Dental Prostheses":ti,ab,kw,de OR "Surgical Dental Prosthesis":ti,ab,kw,de OR "Dental Prosthesis, Surgical":ti,ab,kw,de OR "Dental Implants, Mini":ti,ab,kw,de OR "Dental Implant, Mini":ti,ab,kw,de OR "Mini Dental Implant":ti,ab,kw,de OR "Mini Dental Implants":ti,ab,kw,de
	#5	"tooth implantation"/exp
	#6	"Dental Implantation":ti,ab,kw,de OR "Implantation, Dental":ti,ab,kw,de OR "Implantation, Dental Prosthesis":ti,ab,kw,de OR "Dental Prosthesis Implantations":ti,ab,kw,de OR "Prosthesis Implantation, Dental":ti,ab,kw,de OR "Dental Prosthesis Implantation":ti,ab,kw,de OR "Dental Implant Therapy":ti,ab,kw,de OR "Dental Implant Therapies":ti,ab,kw,de OR "Implant Therapy, Dental":ti,ab,kw,de OR "Therapy, Dental Implant":ti,ab,kw,de
	#7 Combine	#1 OR #2 OR #3 OR #4 OR #5 OR #6
	#8	"enamel matrix proteins":ti,ab,kw,de OR "enamel matrix derivatives":ti,ab,kw,de OR "EMDOGAIN":ti,ab,kw,de OR "enamel matrix derivative":ti,ab,kw,de
	#9	"enamel protein"/exp
	#10	"Dental Enamel Proteins":ti,ab,kw,de OR "Enamel Proteins, Dental":ti,ab,kw,de OR "Proteins, Dental Enamel":ti,ab,kw,de
	#11 Combine	#8 OR #9 OR #10
	#12 Combine	#7 AND #11
	#13 Limit	#12 AND [randomized controlled trial]/lim
Cochrane Library	#1	[mh "Peri-Implantitis"]
	#2	"Peri-Implantitis":ti,ab,kw OR "Peri-Implantitides":ti,ab,kw OR "Peri Implantitis":ti,ab,kw OR "Periimplantitis":ti,ab,kw OR "Periimplantitides":ti,ab,kw OR "peri-implant disease":ti,ab,kw OR "peri-implant diseases":ti,ab,kw OR "Peri-implant":ti,ab,kw OR "peri-implant mucositis":ti,ab,kw
	#3	[mh "Dental Implants"]
	#4	"Dental Implants":ti,ab,kw OR "Dental Implant":ti,ab,kw OR "Implant, Dental":ti,ab,kw OR "Implants, Dental":ti,ab,kw OR "Dental Prostheses, Surgical":ti,ab,kw OR "Prostheses, Surgical Dental":ti,ab,kw OR "Prosthesis, Surgical Dental":ti,ab,kw OR "Surgical Dental Prostheses":ti,ab,kw OR "Surgical Dental Prosthesis":ti,ab,kw OR "Dental Prosthesis, Surgical":ti,ab,kw OR "Dental Implants, Mini":ti,ab,kw OR "Dental Implant, Mini":ti,ab,kw OR "Mini Dental Implant":ti,ab,kw OR "Mini Dental Implants":ti,ab,kw
	#5	[mh "Dental Implantation"]
	#6	"Dental Implantation":ti,ab,kw OR "Implantation, Dental":ti,ab,kw OR "Implantation, Dental Prosthesis":ti,ab,kw OR "Dental Prosthesis Implantations":ti,ab,kw OR "Prosthesis Implantation, Dental":ti,ab,kw OR "Dental Prosthesis Implantation":ti,ab,kw OR "Dental Implant Therapy":ti,ab,kw OR "Dental Implant

		Therapies":ti,ab,kw OR "Implant Therapy, Dental":ti,ab,kw OR "Therapy, Dental Implant":ti,ab,kw
	#7 Combine	#1 OR #2 OR #3 OR #4 OR #5 OR #6
	#8	"enamel matrix proteins":ti,ab,kw OR "enamel matrix derivatives":ti,ab,kw OR "EMDOGAIN":ti,ab,kw OR "enamel matrix derivative":ti,ab,kw
	#9	[mh "Dental Enamel Proteins"]
	#10	"Dental Enamel Proteins":ti,ab,kw OR "Enamel Proteins, Dental":ti,ab,kw OR "Proteins, Dental Enamel":ti,ab,kw
	#11 Combine	#8 OR #9 OR #10
	#12 Combine	#7 AND #11

Supplementary Table S2. Manual searches.

Category		References
Manual searches	1.	Regidor, E. et al., *Enamel Matrix Derivative in the Reconstructive Surgical Therapy of Peri-Implantitis: A Randomized Clinical Trial.* J Periodontal Res, 2025.
	2.	Faramarzi, M. et al., *Microbiological and clinical effects of enamel matrix derivative and sustained-release micro-spherical minocycline application as an adjunct to non-surgical therapy in peri-implant mucosal inflammation.* J Korean Assoc Oral Maxillofac Surg, 2015. 41(4): p. 181-9.

Supplementary Table S3. Excluded studies from full-text reading.

Reasons for exclusion	References
No relevant outcomes	1. Isrctn. Treatment of peri-implantitis with allografts and enamel proteins. https://trialsearchwhoint/Trial2aspx?TrialID=ISRCTN15000657. 2023.<br>2. Actrn. Enamel Matrix Derivative (EMDOGAIN) for the treatment of gum recession and dental implant infection/ridge management. https://trialsearchwhoint/Trial2aspx?TrialID=ACTRN12619000062123. 2019.<br>3. Nct. Regenerative Surgical Treatment of Peri-implantitis. https://clinicaltrialsgov/show/NCT02500654. 2015.<br>4. Irct201311103690N. Effects of minocycline and emdogain in the treatment of peri-implant mucosal inflammation. https://trialsearchwhoint/Trial2aspx?TrialID=IRCT201311103690N4. 2014.<br>5. Irct2013060113543N. the effect of emdogain on peri-implant mucosal inflammation (PIMI). https://trialsearchwhoint/Trial2aspx?TrialID=IRCT2013060113543N1. 2013.
No response received	1. Isrctn. Treatment of peri-implant diseases with enamel matrix proteins. https://trialsearchwhoint/Trial2aspx?TrialID=ISRCTN18159776. 2021.

Supplementary Table S4. Risk of bias.

Study	D1: Randomization process	D2: Deviations from the intended interventions	D3: Missing outcome data	D4: Measurement of the outcome	D5: Selection of the reported result
Faramarzi et al., (2015) [10]	Low risk: “Patients were randomized using a web-based randomization software program (Research Randomizer; http://www.randomizer.org)[20] and then randomly divided into one control group and two test groups.” (Page 183).	Low risk: No deviation from the intended intervention has been reported.	Low risk: Nearly all data for outcome were available.	Low risk: “Each variable was examined on a subject level. The Kolmogorov- Smirnov test was used to determine the normality of data. Median values and interquartile range were estimated for each variable during various assessment intervals of the study (baseline, two weeks, and three months after intervention)” (Page 183).	Some concerns: No information on whether the research results were analyzed before the unblinding of the intervention outcome data.
Isehed (2016) [16]	Low risk: “Patients were randomly allocated to treatment with or without EMD using a pre-defined statistical ensured box randomization.” (Page 864).	Low risk: No deviation from the intended intervention has been reported.	Low risk: Nearly all data for outcome were available.	Low risk: “The examiners of the clinical and radiology registrations were blinded, and the surgeon was blinded until it was time to apply EMD.” (Page 864).	Some concerns: No information on whether the research results were analyzed before the unblinding of the intervention outcome data.
Kashefimehr (2017) [17]	Some concerns: “They were then assigned randomly to receive one of the following treatment protocols by a single clinician (AM), under local anesthesia.” Page 2381).	Low risk:. No deviation from the intended intervention has been reported.	Low risk: Nearly all data for outcome were available.	Low risk: “The calibrated examiner (AK), blinded to the interventions and patients’ assignment, assessed the following variables before (baseline) and 3 months after	Some concerns: No information on whether the research results were analyzed before the unblinding of the intervention outcome data.

				treatment.” (Page 2382).	
Isehed (2018) [13]	Low risk: “Patients were randomized using a web-based randomization software program (Research Randomizer; http://www.randomizer.org)[20] and then randomly divided into one control group and two test groups.” (Page 183).	Low risk: No deviation from the intended intervention has been reported.	Low risk: Nearly all data for outcome were available.	Low risk: “Each variable was examined on a subject level. The Kolmogorov- Smirnov test was used to determine the normality of data. Median values and interquartile range were estimated for each variable during various assessment intervals of the study (baseline, two weeks, and three months after intervention)” (Page 183).	Some concerns: No information on whether the research results were analyzed before the unblinding of the intervention outcome data.
Regidor et al., (2025) [18]	Low risk: “Patients were randomly allocated to treatment with or without EMD using a pre-defined statistical ensured box randomization.” (Page 864).	Low risk: No deviation from the intended intervention has been reported.	Low risk: Nearly all data for outcome were available.	Low risk: “The examiners of the clinical and radiology registrations were blinded, and the surgeon was blinded until it was time to apply EMD.” (Page 864).	Some concerns: No information on whether the research results were analyzed before the unblinding of the intervention outcome data.
